# Human nucleolar protein Nop52 (RRP1/NNP-1) is involved in site 2 cleavage in internal transcribed spacer 1 of pre-rRNAs at early stages of ribosome biogenesis

**DOI:** 10.1093/nar/gkv470

**Published:** 2015-05-12

**Authors:** Harunori Yoshikawa, Hideaki Ishikawa, Keiichi Izumikawa, Yutaka Miura, Toshiya Hayano, Toshiaki Isobe, Richard J. Simpson, Nobuhiro Takahashi

**Affiliations:** 1 Department of Applied Life Science, Graduate School of Agriculture, Tokyo University of Agriculture and Technology, 3-5-8 Saiwai-cho, Fuchu-shi, Tokyo, 183-8509, Japan; 2 Centre for Gene Regulation & Expression, College of Life Sciences, University of Dundee, Dow Street, Dundee, DD1 5EH, UK; 3 Core Research for Evolutional Science and Technology (CREST), Japan Science and Technology Agency (JST), Sanbancho 5, Chiyoda-ku, Tokyo, 102-0075, Japan; 4 Department of Chemistry, Graduate School of Sciences and Engineering, Tokyo Metropolitan University, 1-1 Minamiosawa, Hachiouji-shi, Tokyo 192-0397, Japan; 5 La Trobe Institute for Molecular Science (LIMS), LIMS Building 1, Room 412 La Trobe University, Bundoora Victoria 3086, Australia

## Abstract

During the early steps of ribosome biogenesis in mammals, the two ribosomal subunits 40S and 60S are produced via splitting of the large 90S pre-ribosomal particle (90S) into pre-40S and pre-60S pre-ribosomal particles (pre-40S and pre-60S). We previously proposed that replacement of fibrillarin by Nop52 (RRP1/NNP-1) for the binding to p32 (C1QBP) is a key event that drives this splitting process. However, how the replacement by RRP1 is coupled with the endo- and/or exo-ribonucleolytic cleavage of pre-rRNA remains unknown. In this study, we demonstrate that RRP1 deficiency suppressed site 2 cleavage on ITS1 of 47S/45S, 41S and 36S pre-rRNAs in human cells. RRP1 was also present in 90S and was localized in the dense fibrillar component of the nucleolus dependently on active RNA polymerase I transcription. In addition, double knockdown of XRN2 and RRP1 revealed that RRP1 accelerated the site 2 cleavage of 47S, 45S and 41S pre-rRNAs. These data suggest that RRP1 is involved not only in competitive binding with fibrillarin to C1QBP on 90S but also in site 2 cleavage in ITS1 of pre-rRNAs at early stages of human ribosome biogenesis; thus, it is likely that RRP1 integrates the cleavage of site 2 with the physical split of 90S into pre-40S and pre-60S.

## INTRODUCTION

The ribosome, which consists of four ribosomal RNAs (rRNAs) and ∼80 ribosomal proteins (r-proteins), is essential for protein synthesis in the cell. The mammalian ribosome (80S) is composed of a large 60S subunit (28S, 5.8S, 5S rRNAs and ∼60 r-proteins) and a small 40S subunit (18S rRNA and ∼20 r-proteins). Synthesis of the two subunits begins with transcription of a large ribosomal RNA precursor (47S pre-rRNA) by RNA polymerase I in the fibrillar centres (FC) or at the boundary between the FC and the dense fibrillar component (DFC) in the nucleolus, which is assembled by numerous small nucleolar RNAs (snoRNAs), non-ribosomal proteins (called *trans-*acting factors) and r-proteins to form a large 90S particle (1–3). The four-spacer regions designated as 5′ external transcribed spacer (5′ ETS), internal transcribed space (ITS) 1, ITS2 and 3′ ETS in 47S pre-rRNA are removed as ribosome biogenesis goes on. An endonucleolytic cleavage of ITS1 is believed to be one of the events that is essential for this splitting. Following this cleavage, pre-40S and pre-60S are physically separated, undergo pre-rRNA processing and assembly of r-proteins independently from each other and move from the nucleolus to the nucleoplasm accordingly and eventually to the cytoplasm, where protein synthesis takes place (4). However, whether the ITS1 cleavage and the physical separation of pre-40S from pre-60S particles in 90S are regulated independently from each other is not known. Previously, we showed evidence that the interaction of C1QBP with fibrillarin (FBL) forms a bridge between pre-40S and pre-60S in 90S after the cleavage of ITS1 and is disrupted by the competition of RRP1 with FBL for binding to C1QBP, which causes physical separation of 90S into pre-40S and pre-60S (5). Thus, RRP1 seems to have essential roles in ribosome biogenesis. However, the involvement of RRP1 in pre-rRNA processing remains unknown.

RRP1 is one of two human homologs of yeast Rrp1p, which is present in the 66S pre-ribosome (the yeast counterpart of human pre-60S) and is required for 27S pre-rRNA processing to 25S and 5.8S rRNAs via ITS2 cleavage (6) and for production of the 60S subunit in yeast cells (7,8). Thus, RRP1 is believed to be a factor that is involved in the late stages of ribosome biogenesis. RRP1 is involved in nucleogenesis, localizes mainly in the nucleolus and shuttles between the nucleolus and the pre-nucleolar body, which forms on the chromosome surface and participates in the delivery of the processing machinery at the site of pre-rRNA processing (9–11).

Another human homolog of yeast Rrp1p, RRP1B, is associated with pre-60S (12), is involved in recruitment of protein phosphatase 1 to pre-60S and is suggested to be the functional ortholog of yeast Rrp1p (12). Despite these reports, however, depletion of RRP1B induces only a small effect on pre-rRNA processing and no effect on pre-ribosomal particle assembly (12). In addition, there have been many other reports of the roles that RRP1B has in cellular functions other than ribosome biogenesis, including those in susceptibility for cancer progression and metastasis (13), in modulation of transcription and chromatin structure (14,15) and in E2F-mediated apoptosis (16). Thus, involvement of RRP1B in human ribosome biogenesis is controversial. Our previous results suggest that RRP1, but not RRP1B, may be the functional ortholog of Rrp1p and may participate in human ribosome biogenesis.

Here we provide evidence that RRP1 is involved in the cleavage at site 2 in ITS1 of pre-rRNAs at the early nucleolar stages of human ribosome biogenesis.

## MATERIALS AND METHODS

### Cell culture

HeLa or 293T cells were cultured in DMEM (Sigma) supplemented with 10% heat-inactivated fetal bovine serum, streptomycin (0.1 mg/ml) and penicillin G (100 U/ml) at 37°C in an incubator under 5% CO_2_.

### Plasmid DNA construction

A previous report described the construction of the FLAG-RRP1 expression vector (17). A small interference RNA (siRNA)-resistant RRP1 expression vector was constructed by site-directed mutagenesis, which introduced four silent mutations within the *RRP1* sequence (g215a, a218g, a221g, t222c) to disrupt the target sequence of the stealth siRNA by polymerase chain reaction using the FLAG-RRP1 expression vector as a template. This construct was verified by DNA sequencing.

### RNA interference

HeLa or 293T cells were cultured in 35-mm dishes until they were 70% confluent and then were transfected with 5 μl Lipofectamine RNAiMAX and 100 pmol stealth siRNA (Invitrogen). For co-depletion analysis of RRP1 and XRN2, the cells were transfected with 6 μl Lipofectamine RNAiMAX and a mixture of 60 pmol stealth siRNA targeting *RRP1* mRNA and 60 pmol stealth siRNA targeting *XRN2* mRNA. The following stealth siRNA sequences were used: 5′-CCAGGAAGAAUUAGGAAGGACUAUU-3′ and 5′-AAUAGUCCUUCCUAAUUCUUCCUGG-3′ for RRP1, 5′-GAGAGGAGCAUUGAUGACUGGGUUU-3′ and 5′-AAACCCAGUCAUCAAUGCUCCUCUC-3′ for XRN2, and 5′-CCAAGAAAUUAAAGGCAGGUGGAUU-3′ and 5′-AAUCCACCUGCCUUUAAUUUCUUGG-3′ for a negative control (scRNA). For rescue of the RRP1 knockdown phenotype, HeLa cells in 35-mm dishes were co-transfected with 100 pmol stealth siRNA and 2.5 μg either siRNA-resistant RRP1 expression vector or control vector pcDNA3.1(+) using 5 μl Lipofectamine 2000. The transfected cells were transferred to new dishes 24 h after the transfection and were cultured for an additional 24 h before being used for further analyses.

### Cell fractionation and sucrose density gradient ultracentrifugation

Subconfluent HeLa or 293T cells were collected from four 90-mm dishes, lysed by vortexing for 10 s with 1 ml of cytosol extract buffer (16.7 mM Tris-HCl, pH 8.0; 50 mM NaCl; 1.67 mM MgCl_2_) containing 0.1% Triton X-100 and 1 mM PMSF, incubated for 5 min on ice and centrifuged at 1000 × *g* for 5 min (all centrifugations at 4°C). To fully remove the cytoplasmic constituents, the nuclei were suspended again with 1 ml of the cytosol extract buffer and collected by centrifugation. Pre-ribosome isolation and sucrose density gradient ultracentrifugation were performed as described (18,19). Briefly, the prepared pre-ribosomal (PR) fraction (200 μg/500 μl) was overlaid on 4.5 ml of a 10–40% (w/w) sucrose gradient made in NEB (10 mM Tris-HCl, pH 8.0, 10 mM NaCl, 10 mM EDTA) with 1 mM DTT and 0.01% Genapol C-100 (Sigma). The gradient was centrifuged at 45 000 rpm (average ∼162 500 × *g*) for 100 min at 4°C in a Beckman MLS50 rotor and fractionated through tandem connection with the Gradient Fractionator (BRANDEL) and BioLogic LP system (Bio-Rad), which continuously measured the absorbance at 254 nm. Proteins in each fraction were precipitated with 10% trichloroacetic acid and 10 μg of bovine serum albumin. To extract RNAs, each fraction was treated with 0.1 mg/ml proteinase K, 1% SDS (sodium dodecyl sulfate) and 15 mM EDTA for 1 h at 42°C, extracted with phenol-chloroform and precipitated with isopropanol containing 5 μl of 0.25% linear acrylamide.

### RNA analysis

Metabolic [5,6-^3^H]uridine labeling was performed as described (5). For northern blot analysis, 2 μg of total RNA extracted by Trizol reagent (Invitrogen) was electrophoresed on a 0.7% agarose/formaldehyde gel and transferred to a Hybond-N+ (GE Healthcare), which was subsequently dried, UV cross-linked and hybridized to biotin-labeled DNA oligonucleotide probes at 50°C overnight in PerfectHyb Plus hybridization buffer (Sigma) (20). The hybridized membrane was washed sequentially with 2× SSC containing 0.1% SDS for 5 min at 25°C, 0.5× SSC containing 0.1% SDS for 20 min at 50°C and 0.1× SSC containing 0.1% SDS for 20 min at 25°C. The hybridized RNA was detected using a Chemiluminescent Nucleic Acid Detection Module kit (Thermo Scientific). RNAs were detected using a LAS4000 image analyzer and quantified using MultiGauge software (Fujifilm) (21). The sequences of oligonucleotides used as probes were previously described (5,18), except for the 3′ETS probe (5′-CTCCCAAACCACGCTCCCCGG-3′) to nt 220–240 of the 3′ETS region. Probes were 3′-end labeled using the Biotin 3′-End DNA Labeling kit (Thermo Scientific).

### Antibodies

The antibody sources and dilution ratios used in this study were as follows: rabbit polyclonal anti-RRP1 (GeneTex, 115107; 1:1000 for immunoblot analysis [IB], 1:100 for immunofluorescence staining [IF]), rabbit polyclonal anti-FBL (H-140) (Santa Cruz Biotechnology, 1:1000 for IB), mouse monoclonal anti-FBL (Cytoskeleton, 1:2000 for IF), mouse monoclonal anti-UBF (F-9) (Santa Cruz Biotechnology, 1:50 for IF), mouse monoclonal anti-GAPDH (Ambion, 1:10 000 for IB), goat polyclonal anti-U2AF65 (N-14) (Santa Cruz Biotechnology, 1:1000 for IB), goat polyclonal anti-LaminB (M-20) (Santa Cruz Biotechnology, 1:1000 for IB), rabbit polyclonal anti-B23 (C-19) (Santa Cruz Biotechnology, 1:1000 for IB), mouse monoclonal anti-B23 (Zymed, 1:400 for IF), rabbit polyclonal anti-XRN2 (Bethyl Laboratories, 1:10 000 for IB), rabbit polyclonal anti-BOP1 (GeneTex, 1:1000 for IB), rabbit polyclonal anti-RBM28 (GeneTex, 1:1000 for IB), rabbit polyclonal anti-NNP-1B (RRP1B) (K-19) (Santa Cruz Biotechnology, 1:1000 for IB), goat polyclonal anti-C1QBP (Santa Cruz Biotechnology, 1:2000 for IB). For immunofluorescence staining, the following secondary antibodies were used: FITC-conjugated anti-rabbit IgG (American Qualex, 1:200) and Cy3-conjugated anti-mouse IgG (Sigma, 1:200). For enzyme chemiluminescence labeling, anti-mouse IgG (Cappel, 1:10 000), anti-rabbit IgG (Sigma, 1:10 000), anti-goat IgG (Sigma, 1:10 000) and anti-rabbit IgG EasyBlot (GeneTex, 1:1000) were used as horseradish peroxidase-conjugated antibodies.

### Immunoprecipitation and Immunoblotting

Nuclei extracted from intact 293T cells as described above were lysed by vortexing for 10 s with 1 ml of lysis buffer (50 mM Tris-HCl, pH 8.0; 150 mM NaCl) containing 0.5% IGEPAL CA-630 (Sigma), 1 mM PMSF and 20 U SUPERasin (Ambion), incubated for 10 min on ice and centrifuged at 12 000 × *g* for 10 min at 4°C. For immunoprecipitation of endogenous protein complexes, the prepared nuclear lysate (1.5 mg) was incubated with 3 μg of rabbit normal IgG (Active Motif) or rabbit anti-RRP1 (GeneTex, 115107) for 4 h at 4°C. The antibody-bound RRP1-associated protein complexes were incubated with 10 μl of Dynabeads Protein G (Invitrogen) for 1 h at 4°C, and then the complex-associated beads were washed five times with lysis buffer containing 0.5% IGEPAL CA-630, once with lysis buffer, and were eluted using 2% (w/v) SDS sample buffer. Immunoblotting was performed as described (20,21) using an EasyBlot kit (GeneTex). Signal was detected using Luminol/Enhancer solution and Stable Peroxide solution (Thermo Scientific) as a substrate for peroxidase and using an LAS4000 image analyzer.

### Immunofluorescence staining

Immunofluorescence staining was performed as reported (5) using IMMUNO SHOT immunostaining (CosmoBio) during incubation with appropriate primary and secondary antibodies.

## RESULTS

### RRP1 is present in both pre-60S and 90S particles

**Figure 1.**
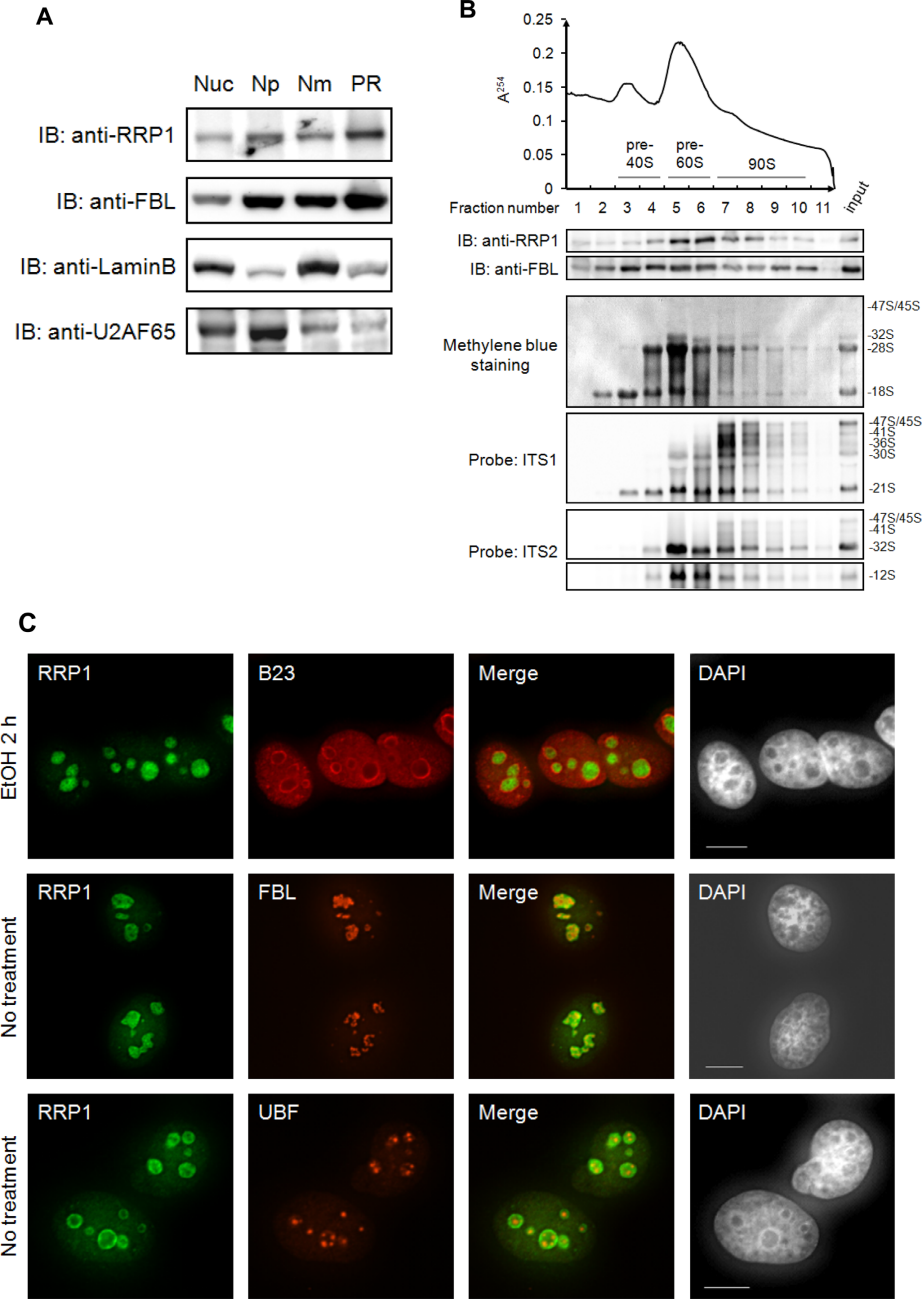

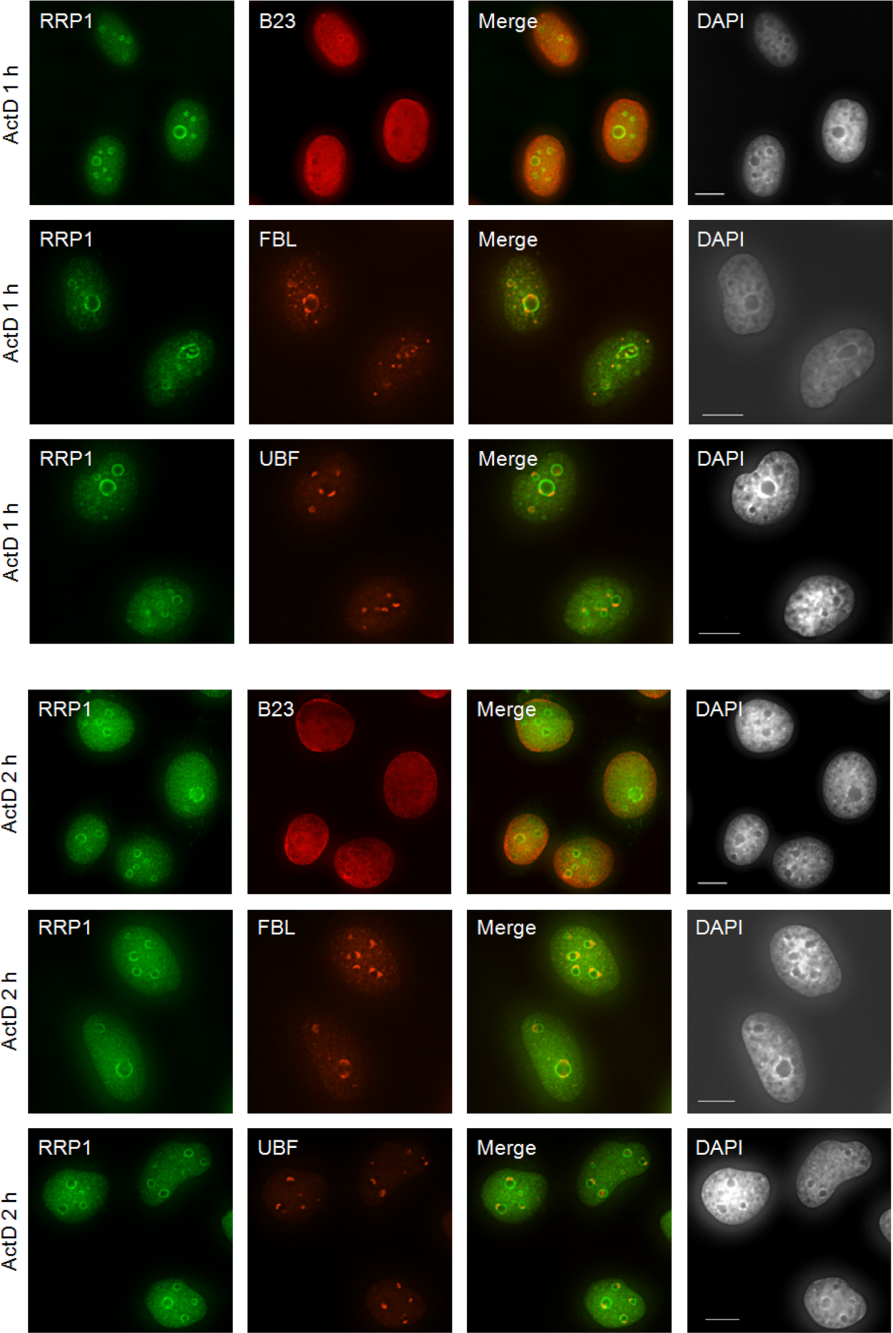

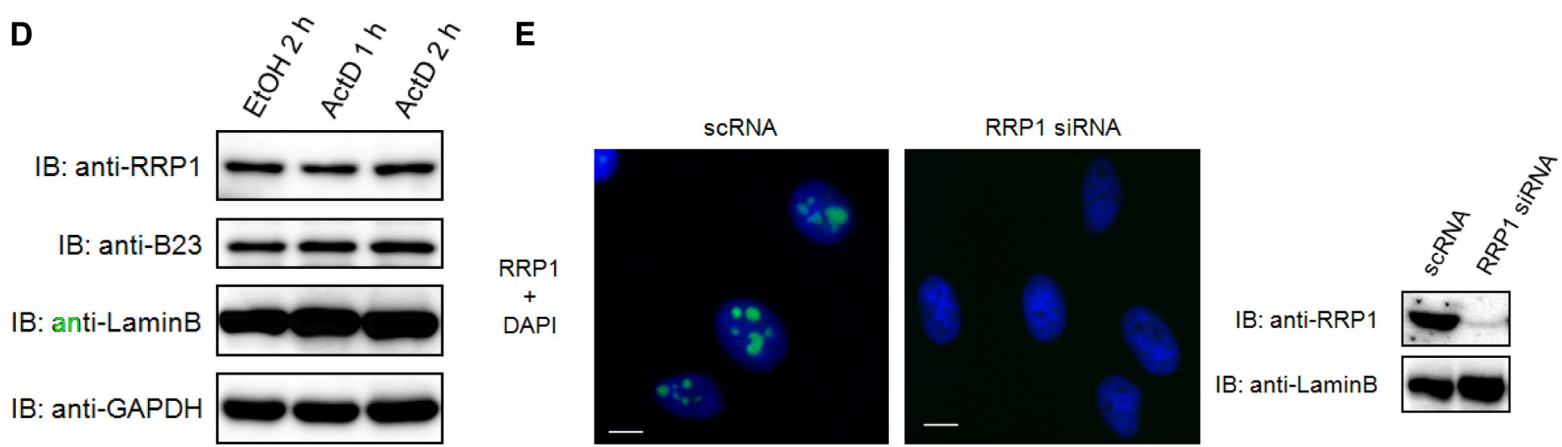
RRP1 is a part of nucleolar pre-ribosomal particles. (**A**) Equivalent amounts (10 μg) of total nuclear extract (Nuc), nucleoplasmic (Np), nuclear matrix (Nm) and pre-ribosomal (PR) fraction prepared from HeLa cells were analyzed by immunoblotting with the antibodies indicated at the left. (**B**) The PR (200 μg) was separated into 11 fractions by ultracentrifugation with a 10–40% sucrose density gradient with continuous monitoring of absorbance at 254 nm (top). Proteins in each fraction were analyzed by immunoblotting (IB) with the antibodies indicated at the left (middle). RNAs extracted from each fraction were separated by agarose gel electrophoresis, transferred to a membrane, stained with methylene blue and hybridized with the biotin-labeled probes indicated at the left (bottom). The pre-rRNA species are assigned at the right. Input: 10 μg of protein and 2 μg of RNA from PR fraction were loaded for IB and agarose gel electrophoresis, respectively. (**C**) HeLa cells were treated with ethanol (EtOH) for 2 h or 0.1 μg/ml ActD for 1 h or 2 h. They were then immunostained using an antibody against RRP1 (green) with B23, FBL or UBF (red) and were stained with DAPI to indicate the nuclei. Bars indicate 10 μm. (**D**) Total protein from cells (2 × 10^4^ cells/lane) treated as above was analyzed by IB with the antibodies indicated at the left. (**E**) HeLa cells treated with scRNA or RRP1 siRNA were immunostained using an antibody against RRP1 (green) with DAPI (left). Bars, 10 μm. Total protein from HeLa cells (20 μg/lane) treated with scRNA or RRP1 siRNA was analyzed by IB with anti-RRP1 or anti-LaminB (right).

Our previous study showed that RRP1 is present in pre-60S fractions and in fractions with higher sedimentation velocity separated by sucrose gradient ultracentrifugation of the nuclear extract (5), suggesting that RRP1 is present not only in pre-60S but also in 90S in human cells. To examine this possibility further, we first used a method of core preribosomal (PR) particle isolation to enrich for 90S that is based on the biochemical subcellular fractionation method, instead of using the co-immunoprecipitation approach, which allows efficient extraction of 90S from yeast cells but not from human cells (19,22). Using this method, RRP1 was enriched in the PR fraction, which also contained high amounts of FBL, a component of the box C/D snoRNP involved in very early stages of ribosome biogenesis (Figure 1A) (23). In contrast, the nucleoplasmic fraction and nuclear matrix fraction were enriched for their marker proteins U2AF65 and LaminB, respectively (Figure 1A). We then separated the PR fraction into 11 fractions by sucrose density ultracentrifugation and detected RRP1 not only in fractions 5 and 6, which also contained 32S pre-rRNA, but also in fractions 7–10, which also contained 47–41S and 36S pre-rRNAs (Figure 1B), suggesting that RRP1 is present both in pre-60S and 90S particles.

We next examined the relationship between RRP1 nucleolar localization and transcription of ribosomal DNA by treating HeLa cells with low levels of actinomycin D (ActD), which inhibits RNA polymerase I-dependent rRNA transcription (24). RRP1 was co-localized partially with FBL in the region inside of the granular component (GC) marker B23 in the nucleolus of interphase cells without ActD treatment (Figure 1C) but RRP1 was not co-localized with UBF in the FC, suggesting that RRP1 is present in a part of the DFC or at the boundary between the FC and DFC in the nucleolus. In contrast, inhibition of rRNA transcription with ActD caused accumulation of RRP1 in the nucleolar periphery and dispersion to the nucleoplasm (Figure 1C). This behavior of RRP1 was somewhat different from that of B23, which dispersed throughout the nucleoplasm without accumulation in the nucleolar periphery, and also different from that of FBL or UBF, which was localized in the nucleolar caps upon ActD treatment (Figure 1C), as previously reported (5,18,25–28). The total amount of RRP1 or B23 in the cell was not affected by ActD treatment (Figure 1D). The specificity of the antibody used was confirmed by the knockdown of RRP1 with siRNA directed to *RRP1* mRNA (RRP1 siRNA), which showed that the nucleolar staining of RRP1 was lost upon treatment with RRP1 siRNA but not upon treatment with scrambled siRNA (scRNA) (Figure 1E). These results indicated that the nucleolar localization of RRP1 was dependent on active rRNA transcription (29). Despite reports that the yeast homolog RRP1 (Rrp1p) is involved only in the processing of ITS2 and the production of the 60S subunit, these data suggest that RRP1 is involved not only in steps occurring at late stages but also in steps at stages occurring at between late and early stages of ribosome biogenesis.

### RRP1 is involved in the cleavages at sites 2 and 4a of human pre-rRNAs

**Figure 2.**
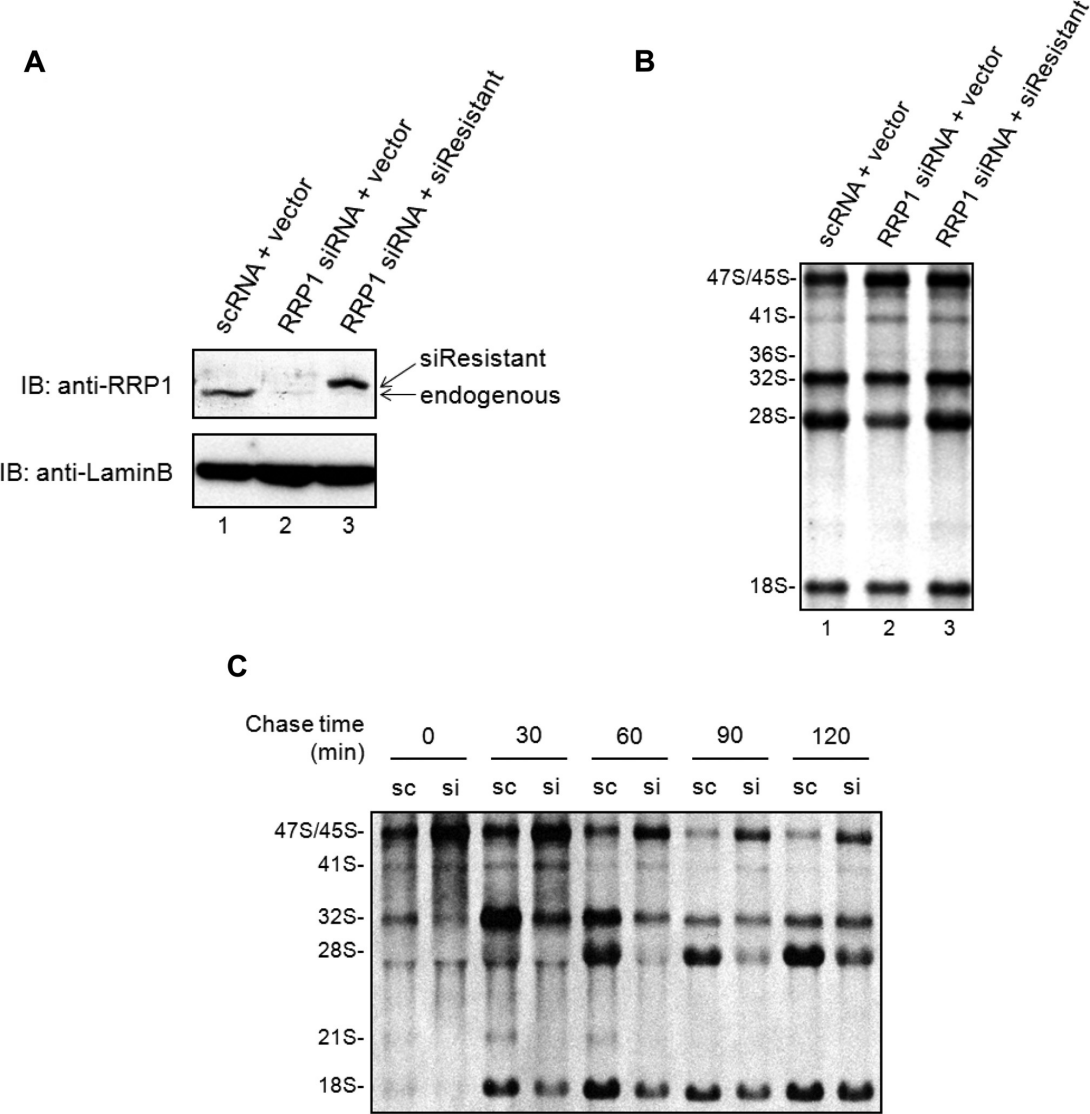
RRP1 is involved in 47S/45S and 32S pre-RNA processing. (**A**) Total protein from HeLa cells (20 μg/lane) transfected with scRNA and empty vector (lane 1: scRNA + vector), RRP1 siRNA and empty vector (lane 2: RRP1 siRNA + vector), or RRP1 siRNA and siRNA-resistant RRP1 expression vector (lane 3: RRP1 siRNA + siResistant) was analyzed by immunoblotting (IB) with anti-RRP1 or anti-LaminB. Endogenous RRP1 and exogenously expressed FLAG-tagged siRNA-resistant RRP1 (siResistant) are indicated. (**B**) HeLa cells treated as above were metabolically labeled with [^3^H]uridine for 2 h. Total RNA was isolated and equal counts per minute (20 000 cpm) per sample were separated on an agarose gel, transferred to a membrane and detected by fluorography. Positions of rRNAs and major precursors are indicated at the left. (**C**) HeLa cells treated with scRNA (sc) or RRP1 siRNA (si) were labeled with [^3^H]uridine for 30 min, cultured in nonradioactive medium containing 0.5 mM nonradioactive uridine for the times indicated and then analyzed as in (B).

To further examine the roles of RRP1 in steps at early stages of ribosome biogenesis, we knocked down *RRP1* mRNA in HeLa cells with siRNA for 48 h and detected pre-rRNA processing by metabolic labeling with [^3^H]uridine for 2 h. Immunoblot analysis showed that RRP1 was depleted effectively in RRP1 siRNA-treated cells (Figure 2A, lanes 1 and 2). The deficiency of RRP1 caused accumulation of 47S/45S and 41S pre-rRNAs and reduction of 32S pre-rRNA and mature 18S and 28S rRNAs (Figure 2B, lanes 1 and 2). These reductions were rescued significantly by transfection with epitope-tagged RRP1 siRNA-resistant plasmid (Figure 2A and B, lanes 2 and 3), indicating that these accumulation and reduction were caused by RRP1 depletion in the cells. In addition, pulse-chase experiments clearly showed that 47S/45S pre-rRNA accumulated and that processing from 47S/45S to 32S pre-rRNAs and 32S pre-rRNA to 28S rRNA was reduced upon knockdown of RRP1 (Figure 2C). Decrease of 18S rRNA production was also clearly seen upon knockdown from as early as 30 min after pulse labeling, consistent with an inhibition of the processing of 47S/45S pre-rRNAs (Figure 2C). These results suggest that RRP1 is involved in the processing of 47S/45S pre-rRNAs at early stages and that of 32S pre-rRNA at late stages of ribosome biogenesis in human cells.

**Figure 3.**
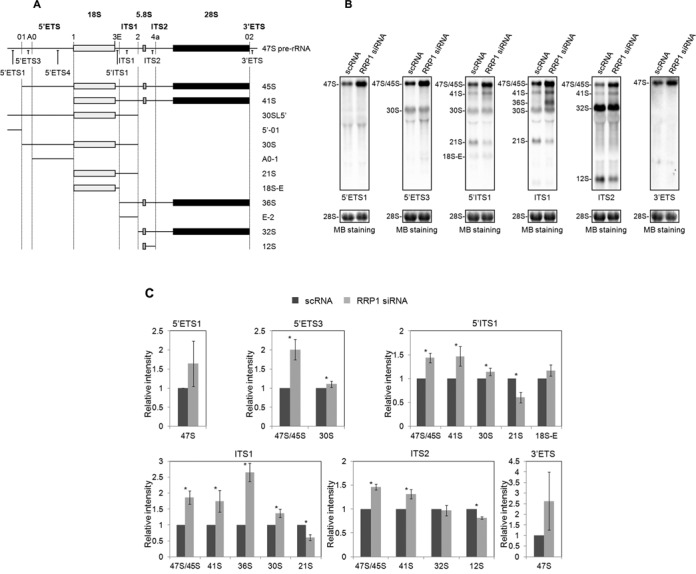
RRP1 is involved in the processing of 5′ ETS, ITS1 and ITS2 regions. (**A**) Structure of the 47S pre-rRNA and pre-rRNA intermediates that were detected by northern blot analysis using the oligonucleotide probes indicated. The cleavage sites are labeled above the 47S pre-rRNA and indicated by vertical dotted lines, and the probe locations are shown below the 47S pre-rRNA. Also, the 18S, 5.8S and 28S mature rRNAs are indicated as light gray, gray and black boxes, respectively. (**B**) Pre-rRNA intermediates isolated from HeLa cells treated with scRNA or RRP1 siRNA were detected by northern blot analysis (top). Oligonucleotide probes shown in (A) are indicated at the bottom of each staining set. 28S rRNA was visualized by methylene blue staining (MB staining) as a loading control (bottom). The pre-rRNA species are assigned at the left of each staining set. (**C**) The staining intensity of each band in each staining set was quantified, first normalized to the amount of 28S rRNA and then graphed relative to the amount of the corresponding band from scRNA-treated cells (bottom). The values are averages (± SD) of three independent experiments. *, *P* < 0.05.

To locate the regions in pre-rRNAs where RRP1 regulates the processing, we carried out northern blot analysis using probes specific for 5′ETS1, 5′ETS3, 5′ITS1, ITS1, ITS2 and 3′ETS before and after knockdown of RRP1 in HeLa and 293T cells (Figure 3A). We first confirmed that the knockdown by siRNA reduced RRP1 to less than 10% relative to LaminB when compared with scRNA-treated HeLa and 293T cells (Supplementary Figure S1). Northern blot analysis revealed that the primary transcripts of 47S pre-rRNA (detected with 5′ETS1 and 3′ETS probes) accumulated by at least 130% relative to 28S methylene blue staining in RRP1-depleted HeLa and 293T cells when compared with control cells (Figure 3B, C and Supplementary Figure S2), indicating that the deficiency of RRP1 delays early cleavage at both site 01 in the 5′ ETS region and site 02 in the 3′ ETS region (Figure 3A). The RRP1-depleted cells also accumulated pre-rRNAs that were indistinguishable between 45S and 47S (47S/45S) (detected with 5′ ETS3, 5′ ITS1, ITS1 and ITS2 probes) (Figure 3B, C and Supplementary Figure S2). In addition, RRP1-depleted cells significantly accumulated 41S (detected with 5′ ITS1, ITS1 and ITS2 probes), 36S (detected with ITS1 probe) and 30S (detected with 5′ ETS3, 5′ ITS1 and ITS1 probes) and had lower amounts of 21S (detected with 5′ ITS1 and ITS1 probes) and 12S (detected with ITS2 probe) pre-rRNAs (Figure 3B, C and Supplementary Figure S2). These results indicated that the site 2 cleavage was severely impaired in RRP1-depleted cells. Although we could detect marginal changes in the 32S pre-rRNA level in RRP1-depleted cells because of its abundance, which caused saturated staining by northern blotting (Figure 3B, C and Supplementary Figure S2), this intermediate was clearly decreased within a specific time window (i.e. 30–60 min after the pulse) in *in vivo* metabolic pulse-labeling experiments (Figure 2B and C). Thus, the production of 32S pre-rRNA was impaired in RRP1-depleted cells, suggesting that RRP1 is involved in cleavage at site 2. Furthermore, the pulse-labeling experiments also suggested that the deficiency of RRP1 impaired the processing of 32S pre-rRNA to 28S rRNA (Figure 2B and C). In this case, RRP1 was involved in the site 4a cleavage. This is also supported by the reduction of 12S pre-rRNA, which is generated through the site 4a cleavage, by depletion of RRP1 (Figure 3B, C and Supplementary Figure S2). The involvement of RRP1 in this cleavage in ITS2 is consistent with that reported for its yeast ortholog, which is required for ITS2 cleavage of 27S pre-rRNA, the yeast counterpart of 36S pre-rRNA (6–8).

### RRP1 regulates site 2 cleavage at the first stage of 47S pre-rRNA processing

**Figure 4.**
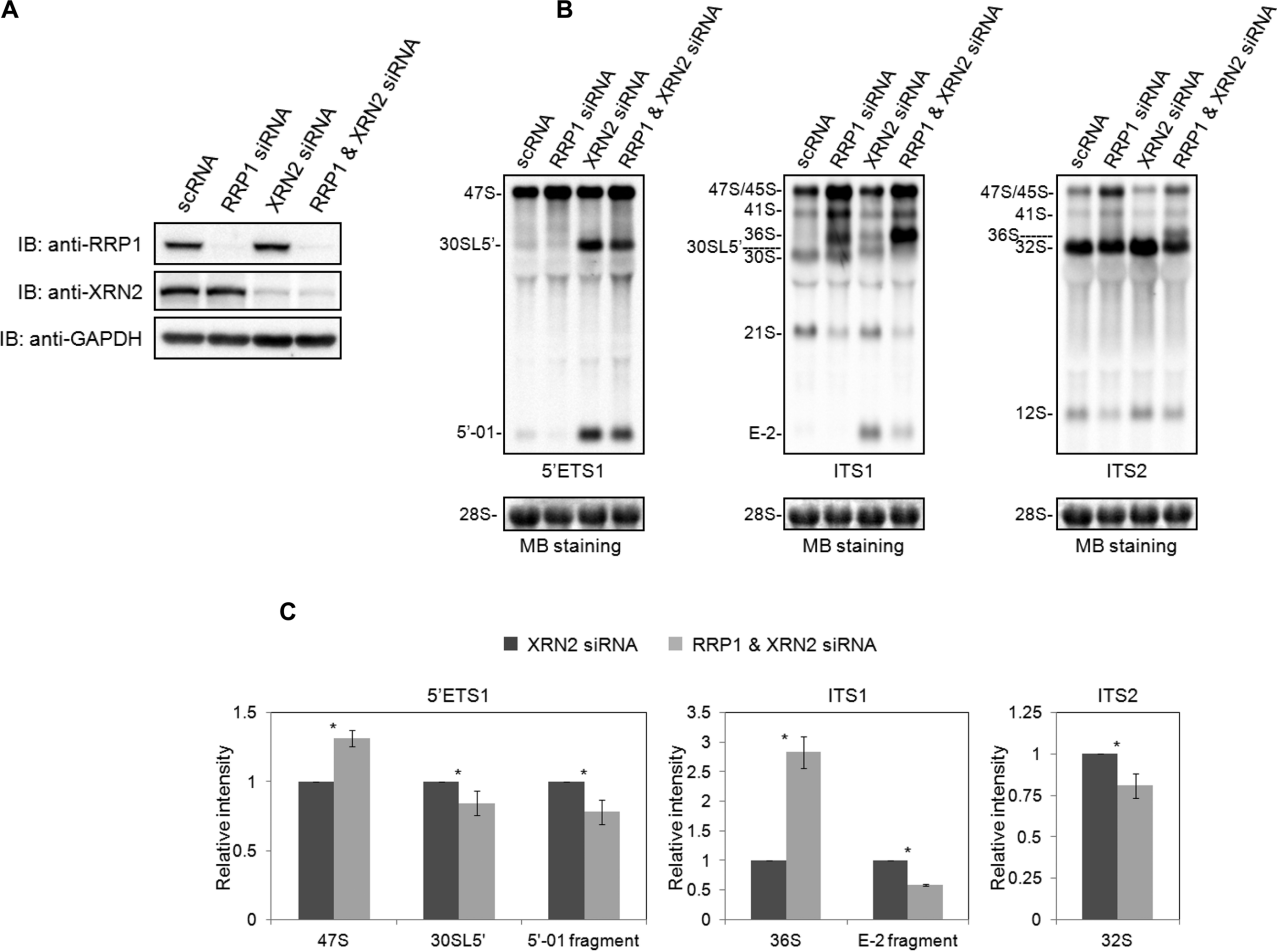
RRP1 accelerates the site 2 cleavage of 47S/45S pre-rRNA. (**A**) Total protein from HeLa cells (20 μg/lane) treated with scRNA, RRP1 siRNA, XRN2 siRNA, or RRP1 and XRN2 siRNA was analyzed by immunoblotting (IB) with the antibodies indicated at the left. (**B**) Pre-rRNA intermediates isolated from HeLa cells treated as in (A) were detected by northern blot analysis using 5′ ETS1, ITS1 and ITS2 probes as indicated at the bottom of each staining set (top). 28S rRNA was visualized by methylene blue staining (MB staining) as a loading control (bottom). The pre-rRNA species are assigned at the left of each staining set. (**C**) The intensities of the 47S, 30SL5′ pre-rRNA and 5′-01 fragment detected by 5′ ETS1, of the 36S pre-rRNA and E-2 fragment detected by ITS1, and of the 32S pre-rRNA detected by ITS2 were quantified, first normalized to the amount of 28S rRNA and then graphed relative to the amount of the corresponding band from XRN2 siRNA-treated cells. The values are averages (± SD) of three independent experiments. *, *P* < 0.05.

The 5′-01 fragment is generated by cleavage at site 01 in the 5′ ETS region, and the E-2 fragment is generated by two endonucleolytic cleavages, at site E and site 2 in the ITS1 region (Figure 3A). Those fragments are degraded by the 5′-to-3′ exonuclease XRN2 in mouse and human cells (30–33). The degradation of those fragments is required for the processing of 36S and 30SL5′, so that depletion of XRN2 causes the accumulation of 36S and 30SL5′ (30-33). We took the advantage of this to determine the involvement of RRP1 in the cleavage at site 2 of 47S pre-rRNA. We first assessed the efficiency of XRN2 siRNA in HeLa cells, both alone and in the presence of RRP1 siRNA (Figure 4A). We confirmed that the knockdown of XRN2 alone caused an accumulation of the E-2 and 5′-01 fragments and 36S, 32S and 30SL5′ pre-rRNAs as reported (Figure 4B) (30–33), whereas E-2, 5′-01 and 30SL5′ were hardly detected upon RRP1 knockdown alone (Figure 4B and C). Therefore, we knocked down both RRP1 and XRN2 and showed that the double knockdown reduced levels of E-2 and 5′-01 fragments and 30SL5′ pre-rRNA relative to methylene blue staining of 28S when compared with those of the XRN2 knockdown alone (Figure 4A, B and C). The double knockdown also reduced the level of 32S pre-rRNA (detected by ITS2) but accumulated its precursor 36S pre-rRNA (detected with ITS1 and ITS2) when compared with that of the XRN2 knockdown alone. These data suggest that those reductions were caused by the suppression of the site 2 cleavage of 47S pre-rRNA. Without cleavage at site 2, 30SL5′ and 32S pre-rRNAs cannot be produced from 47S pre-rRNA. In fact, the knockdown of RRP1 increased 47S pre-rRNA and decreased 32S pre-rRNA (Figures 2C and 3B, and C). Together, these data suggest that RRP1 is required for the site 2 cleavage in concert with the action of XRN2, which has a suppressive effect on both cleavages.

### RRP1 is associated with the site 2 cleavage factors

**Figure 5.**
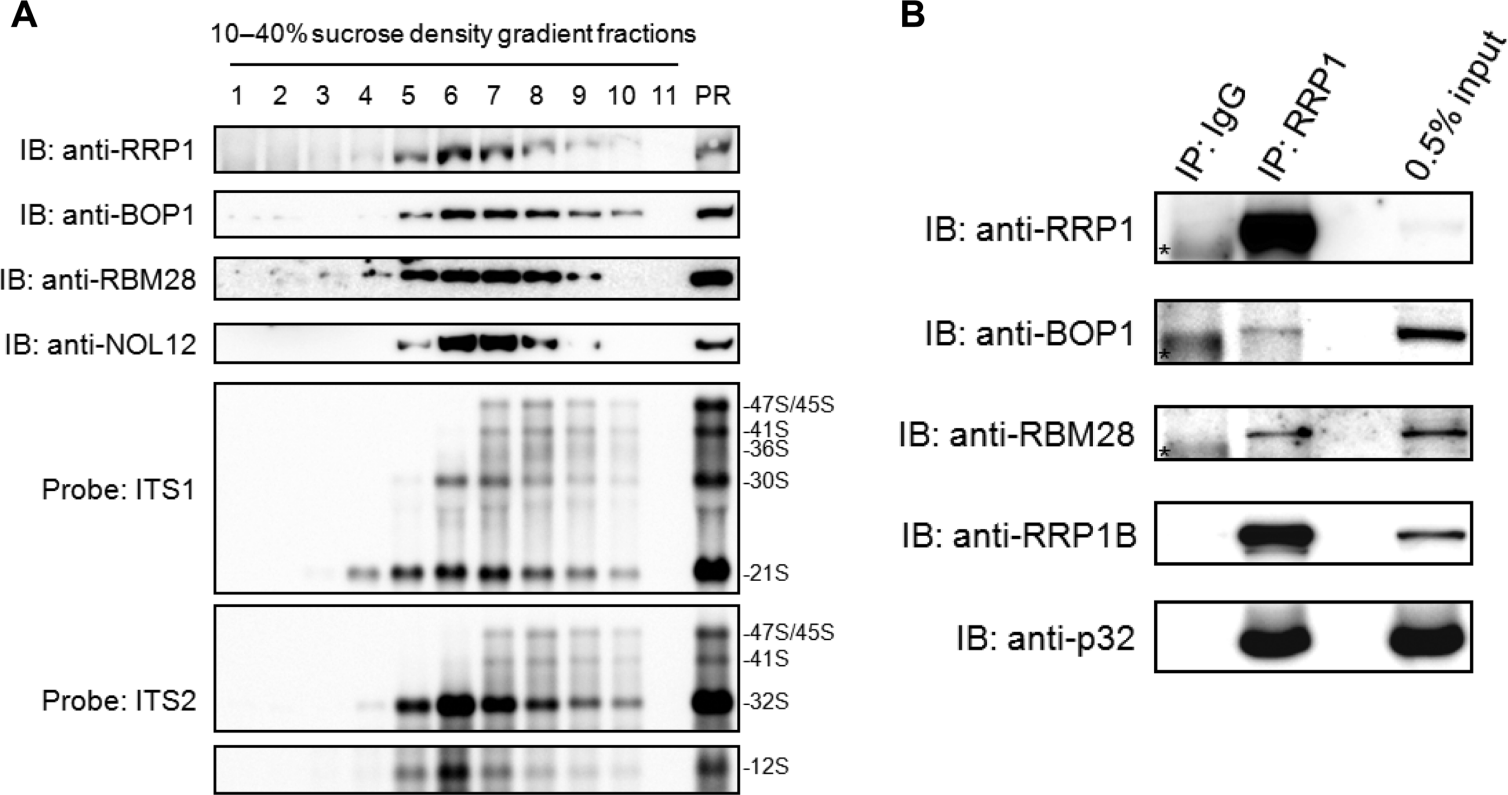
RRP1 co-fractionated and associated with the ITS2 cleavage factors in 90S particles. (**A**) The PR fraction was extracted and fractionated as in Figure 1B. Proteins in each fraction were analyzed by immunoblotting (IB) with the antibodies indicated at the left (top). RNAs extracted from each fraction were separated by agarose gel electrophoresis, transferred to a membrane and hybridized with the biotin-labeled probes indicated at the left (bottom). The pre-rRNA species are assigned at the right. Input: 10 μg of protein and 2 μg of RNA from each PR fraction were loaded for immunoblotting and agarose gel electrophoresis, respectively. (**B**) RRP1 was immunoprecipitated (IP) with anti-RRP1 from the nuclear lysate of 293T cells. Associated proteins were analyzed by immunoblotting with the antibodies indicated at the left. Normal rabbit IgG was used as a negative control for IP. The input control refers to the nuclear lysate used for IP. An asterisk indicates the presence of a non-specific band.

Given the recent report that BOP1, RBM28 and NOL12 are responsible for cleavage at site 2 in the ITS1 region in human cells (32), we examined whether RRP1 was associated with those proteins in the pre-ribosomal particles. Ultracentrifugation analysis of the nucleolar PR fraction showed that RRP1 co-eluted with the three proteins not only in fractions 5 and 6 containing the 32S and 12S pre-RNAs but also in fractions 7–10 containing 47S/45S, 41S, and 36S pre-rRNAs (Figure 5A), suggesting that those four proteins are present in both pre-60S and 90S particles. In addition, immunoprecipitation with anti-RRP1 from the nuclear extract of 293T cells demonstrated that endogenous RRP1 was associated with BOP1 and RBM28 (Figure 5B), suggesting that RRP1 regulates the site 2 cleavage in the ITS1 region in nucleolar 90S particles, presumably with at least BOP1 and RBM28. As the molecular weight of NOL12 and light chains of IgG that we used for immunoprecipitation was almost the same, we could not detect NOL12 clearly in RRP1-assciated protein complex by immunoblot analysis (data not shown). In addition, we detected RRP1B and C1QBP, both of which are reported to associate with RRP1 (5,12,14).

## DISCUSSION

On the basis of homology of RRP1 with yeast Rrp1p, which is required for 27S pre-rRNA processing to 25S and 5.8S rRNAs via ITS2 cleavage (6) and for the production of the 60S subunit in yeast cells (7,8), RRP1 was thought to be a late processing factor involving in 60S production; however, in this study, we demonstrated that RRP1 is also required for early processing of site 2 of 47S/45S, 41S and 36S pre-rRNA (Figure 3A).

**Figure 6.**
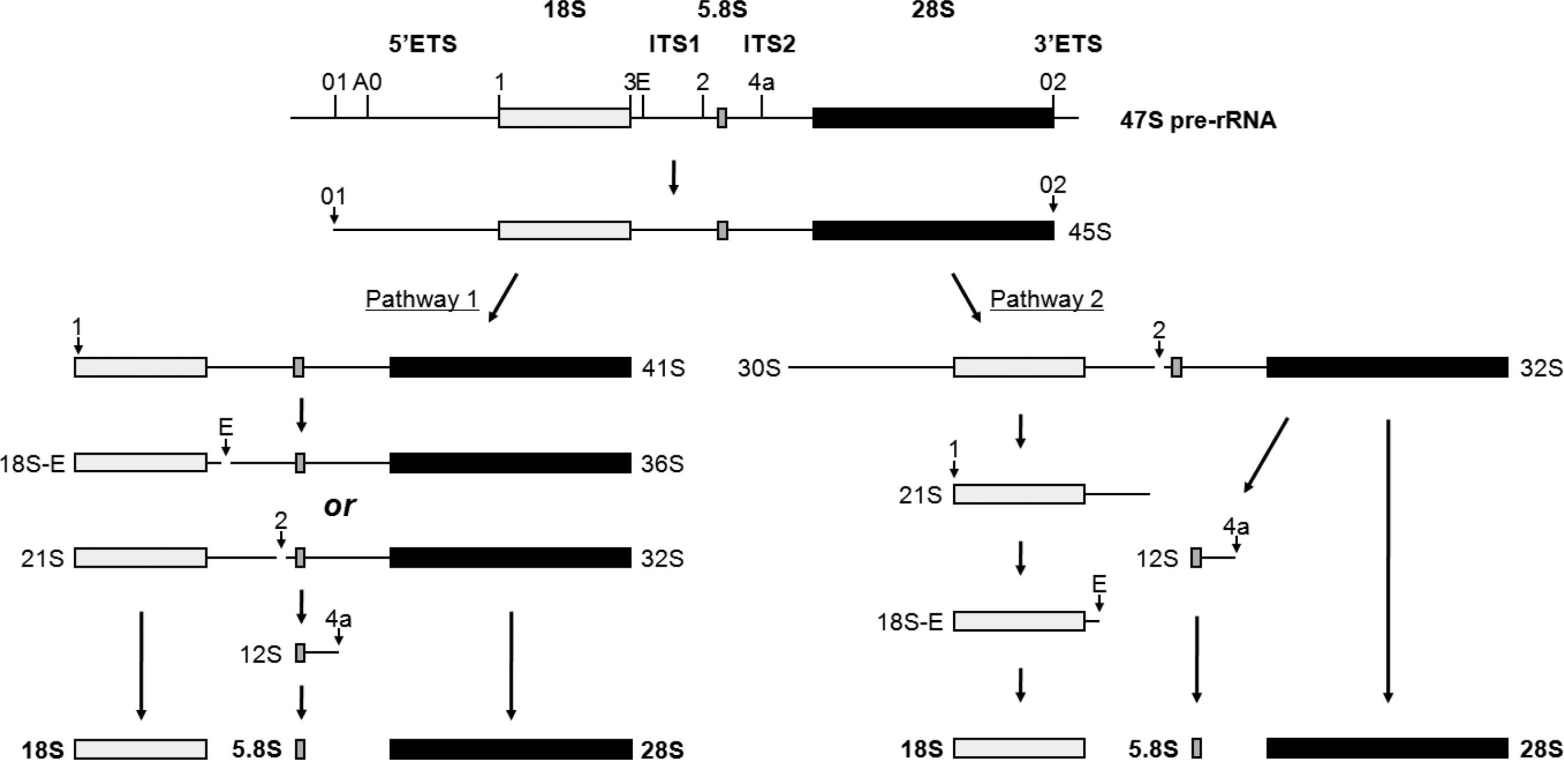
Processing of human pre-rRNA. Structure of the primary rRNA transcript (47S) and flowchart of pre-rRNA processing in human cells (adapted from 34–36). In human cells, two major pathways (pathway 1 and pathway 2) have been proposed and are shown.

In mammalian cells, two major pathways (pathways 1 and 2) are proposed for the processing of 47S pre-rRNA (Figure 6) (34–36). Both pathways start from 45S pre-rRNA that is formed by the removal of the 5′-01 fragment and 3′ETS region from the initial RNA polymerase I-transcript 47S pre-rRNA. In pathway 1, 45S pre-rRNA is first processed into 41S pre-rRNA and the 01–1 fragment of 5′ETS region by cleavage at site 1. The 41S pre-rRNA is further processed either into 18S-E and 36S pre-rRNAs by cleavage at site E or into 21S and 32S pre-rRNAs by cleavage at site 2. In the former case, the 36S pre-rRNA is processed into the E-2 fragment and 32S pre-rRNA, whereas 18S-E is processed into mature 18S rRNA. In the latter case, the 21S pre-RNA is processed into 18S-E and eventually forms 18S rRNA, whereas the 32S pre-rRNA is processed into 12S pre-rRNA and 28S rRNA (Figure 6). Our present data showed that HeLa and 293T cells utilize the former route of pathway 1 for the processing of pre-rRNA under the deficiency of RRP1, which caused accumulation of 41S and 36S pre-rRNAs and reduction of 32S and 12S pre-rRNAs (Figures 2C and 3B, C and Supplementary Figure S2), suggesting that RRP1 is involved in site 2 cleavage of 41S and/or 36S pre-rRNA but not site E cleavage of 41S pre-rRNA and/or 21S pre-rRNA. It seems that site E cleavage dominates when RRP1 is deficient. This is supported by the result that the depletion of RRP1 has no effect on the formation of 18S-E pre-rRNA (Figure 3B, C and Supplementary Figure S2) and by the fact that the level of 36S pre-rRNA is very low under normal conditions in human and mouse cells (31,37,38). Although the reduction of 12S pre-rRNA and 28S rRNA upon knockdown of RRP1 can also be explained by inhibition of the cleavage at site 2 of 36S and 41S pre-rRNAs upstream of the site 4a cleavage in this pathway, RRP1 is also involved in site 4a cleavage as shown by the results of the pulse-chase experiments as well as those of northern blot analysis (Figures 2C and 3B, C and Supplementary Figure S2).

In pathway 2 (Figure 6), 45S or 47S pre-rRNA is first processed into 30S or 30SL5′ pre-rRNA and 32S pre-rRNA by the cleavage at site 2. The 30S or 30SL5′ pre-rRNA is further processed into 21S and/or 18S-E pre-rRNAs by cleavage at site 1, whereas the 32S pre-rRNA is processed into 12S pre-rRNA and 28S rRNA by the cleavage at site 4a (Figure 6). Given that 47S/45S accumulated under RRP1 deficiency, we further tested the possibility that RRP1 was also required for the cleavage at site 2 of 47S/45S pre-rRNA of pathway 2. Because the deficiency of XRN2 causes an accumulation of E-2 and 5′-01 fragments and 30SL5′ pre-rRNA, which is a 30S pre-rRNA with an additional 5′ fragment of 5′ ETS (5′-01 fragment), we examined the effects of double knockdown of XRN2 and RRP1 on the formation of 30SL5′ pre-rRNA and the 5′-01 fragment. The idea behind this experiment was that under the knockdown of XRN2, the E-2 and 5′-01 fragments and 30SL5′ pre-rRNA could be visualized, so the effect of the RRP1 knockdown on these RNAs could be seen clearly by northern blot analysis with the 5′ ETS1 and ITS1 probes. We showed that the double knockdown of RRP1 and XRN2 increased 47S and 47S/45S pre-rRNA levels but reduced 30SL5′ and 32S pre-rRNA levels when compared with those from XRN2 knockdown alone, indicating that RRP1 accelerates the site 2 cleavage of 47S and 47S/45S pre-rRNAs (Figure 4A and B). The data, in turn, suggest that RRP1 regulates the cleavage at site 2 in pathway 2.

The accumulation of 30S pre-rRNA upon the knockdown of RRP1 suggests a possibility that RRP1 is involved in site 1 cleavage (Figure 3B, C and Supplementary Figure S2). Indeed, A0-1 fragment detected by northern blot analysis using 5′ ETS4 probe (Figure 3A) was reduced in RRP1-XRN2-co-depleted cells when compared with that in XRN2-depeleted cells (Supplementary Figure S3). In conjunction with the observed decrease of 21S pre-rRNA upon the knockdown of RRP1 (Figure 3B, C and Supplementary Figure S2), we also suggest that RRP1 is involved in not only site 2 cleavage but also site 1 cleavage in pathway 2 (Figure 6). The involvement of RRP1 in the site 2 and site 1 cleavage is consistent with its presence in 90S as indicated by ultracentrifugation analysis (Figure 1B).

The accumulation of 47S pre-rRNAs under RRP1 deficiency also raises the possibility that RRP1 is involved in the cleavage at the site 01 and site 02 (Figures 2C and 3B, C and Supplementary Figure S2). However, a ratio of 30SL5′ and 5′-01 fragment between the knockdown of XRN2 and the double knockdown of RRP1 and XRN2 is almost the same (Supplementary Figure S4), suggesting that RRP1 is not involved in site 01 cleavage. On the other hand, we observed that the deficiency of RRP1 caused the accumulation of 47S pre-rRNA that was detected by 3′ ETS probe, though abnormal pre-rRNAs extended into 3′ ETS was not detected (22,39); thus, it is possible that RRP1 is involved in site 02 cleavage (Figures 2C and 3B, C and Supplementary Figure S2).

We recently reported that RRP1 is involved in the splitting of FBL-C1QBP-associated 90S particles into RRP1-C1QBP-associated pre-60S particles (5). Our hypothesis is that RRP1 targets C1QBP on the FBL-associated 90S particle and then splits it into pre-60S and pre-40S via the competition between RRP1 and FBL for binding to C1QBP. It is essential to cleave site 2 on the ITS1 region before splitting of the 90S. The present data suggest that RRP1 accelerates the site 2 cleavage in cooperation with, at a minimum, BOP1 and RBM28, which are known to be involved in site 2 cleavage (32). Since mouse Pes1, a component of Pes1–Bop1–WDR12 (PeBoW) complex and Nog1 are involved in site 2 cleavage, it is very interesting to know whether RRP1 also co-operates with those proteins in site 2 cleavage, though the endonuclease that is responsible for the cleavage at site 2 has not been identified (37,40,41). It should be noted here, however, that site 2 cleavage is significantly impaired by depletion of BOP1, RBM28 and NOL12 in human cells, and by depletion of Pes1 and Nog1 in mouse cells, which are known late factors of ribosome biogenesis (32,37). We, therefore, do not exclude a possibility that RRP1 may have roles in site 2 cleavage as just one of the many late processing factors, which exhibit similar phenotypes. Nonetheless, together, the present data strongly support our previous proposal that the replacement of FBL by RRP1 for binding to C1QBP is a key event that drives the physical splitting of 90S into pre-40S and pre-60S. RRP1 is involved not only in this physical separation but also in the cleavage at site 2 in ITS1, both of which are essential to produce the small and large ribosomal subunits in human cells.

## SUPPLEMENTARY DATA

Supplementary Data are available at NAR Online.

SUPPLEMENTARY DATA
