# Supplementary material for: Human nucleolar protein Nop52 (RRP1/NNP-1) is involved in site 2 cleavage in internal transcribed spacer 1 of pre-rRNAs at early stages of ribosome biogenesis
Source: Nucleic Acids Res. 2015 May 12;43(11):5524–36. doi: 10.1093/nar/gkv470 (PMC4477673; doi:10.1093/nar/gkv470)
Supplement: SUPPLEMENTARY DATA [file supp_43_11_5524__index.html]

Human nucleolar protein Nop52 (RRP1/NNP-1) is involved in site 2 cleavage in internal transcribed spacer 1 of pre-rRNAs at early stages of ribosome biogenesis — Human nucleolar protein Nop52 (RRP1/NNP-1) is involved in site 2 cleavage in internal transcribed spacer 1 of pre-rRNAs at early stages of ribosome biogenesis — SUPPLEMENTARY DATA 

# Human nucleolar protein Nop52 (RRP1/NNP-1) is involved in site 2 cleavage in internal transcribed spacer 1 of pre-rRNAs at early stages of ribosome biogenesis

## SUPPLEMENTARY DATA

- SUPPLEMENTARY DATA
