# Supplementary material for: Human nucleolar protein Nop52 (RRP1/NNP-1) is involved in site 2 cleavage in internal transcribed spacer 1 of pre-rRNAs at early stages of ribosome biogenesis
Source: Nucleic Acids Res. 2015 May 12;43(11):5524–36. doi: 10.1093/nar/gkv470 (PMC4477673; doi:10.1093/nar/gkv470)
Supplement: SUPPLEMENTARY DATA [file supp_gkv470_nar-00089-r-2015-File003.pdf]

Supplementary Materials

Human nucleolar protein Nop52 (RRP1/NNP-1) is involved in site 2 cleavage in internal transcribed spacer 1 of pre-rRNAs at early stages of ribosome biogenesis

Harunori Yoshikawa[1,2], Hideaki Ishikawa[1,3], Keiichi Izumikawa[1,3], Yutaka Miura[1], Toshiya Hayano[1], Toshiaki Isobe[3,4] , Richard J Simpson[5] and Nobuhiro Takahashi[1,3,*]

[1]Department of Applied Life Science, Graduate School of Agriculture, Tokyo University of Agriculture and Technology, 3-5-8 Saiwai-cho, Fuchu-shi, Tokyo, 183-8509, Japan

[2]Centre for Gene Regulation & Expression, College of Life Sciences, University of Dundee, Dow Street, Dundee, DD1 5EH, UK

[3]Core Research for Evolutional Science and Technology (CREST), Japan Science and Technology Agency (JST), Sanbancho 5, Chiyoda-ku, Tokyo, 102-0075, Japan

[4]Department of Chemistry, Graduate School of Sciences and Engineering, Tokyo Metropolitan University, 1-1 Minamiosawa, Hachiouji-shi, Tokyo 192-0397, Japan

[5]La Trobe Institute for Molecular Science (LIMS) LIMS Building 1, Room 412 La Trobe University, Bundoora Victoria 3086, Australia

*To whom correspondence should be addressed. Tel: +81-(0)42-367-5709; Fax: +81-(0)42-367-5709; Email: ntakahas@cc.tuat.ac.jp

Present Address: [Toshiya Hayano], Department of Biomedical Sciences, College of Life Sciences, Ritsumeikan University, 1-1-1 Nojihigashi, Kusatsu-shi, 525-8577, Japan

Supplementary Figure 1

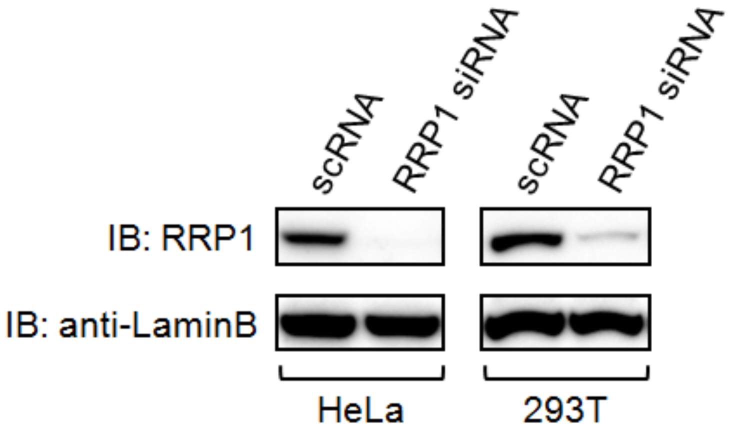

Total protein from HeLa or 293T cells (20 μg/lane) treated with scRNA or RRP1 siRNA was analyzed by immunobloting (IB) with anti-RRP1 or anti-LaminB.

Supplementary Figure 2

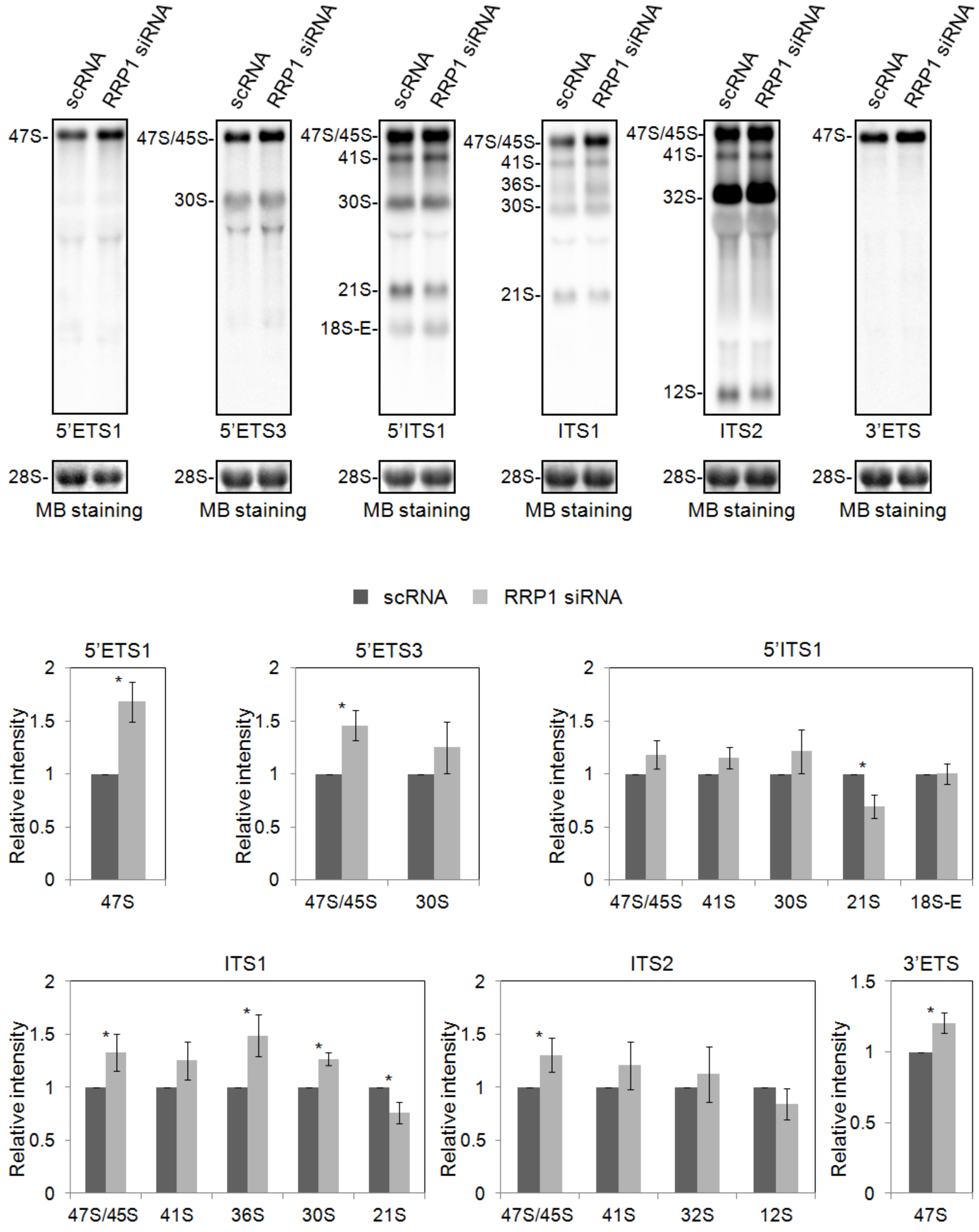

Pre-rRNA intermediates isolated from 293T cells treated with scRNA or RRP1 siRNA were detected by northern blot analysis (top) and quantified (bottom) as in Figure.3B and C.

Supplementary **Figure 3**

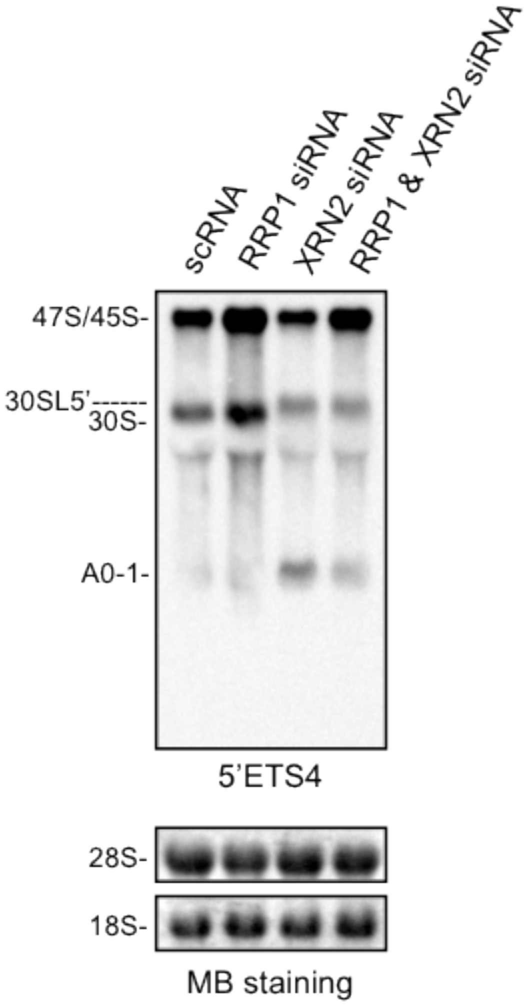

The A0-1 fragment was detected by northern blot analysis with 5'ETS4 probe (Figure. 3A) in XRN2-depleted cells and in RRP1-XRN2-co-depleted cells.

Supplementary Figure 4

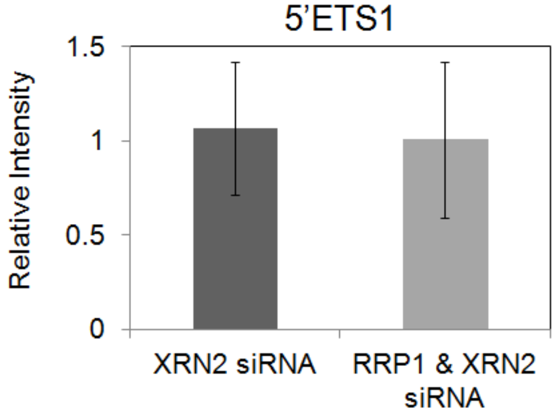

The ratio of the 30SL5' pre-rRNA and 5'-01 fragment detected by 5'ETS1 probe (Figure 4B) are shown for XRN2-depleted cells and RRP1-XRN2-co-depleted cells. The intensities of 30SL5' and 5'-01 were quantified, and normalized to the amount of 28S rRNA. The values are average (± SD) of three independent experiments.
